# Supplementary material for: Scaffold-Free Bone Regeneration Through Collaboration Between Type IV Collagen and FBXL14
Source: J Clin Med. 2025 Oct 11;14(20):7160. doi: 10.3390/jcm14207160 (PMC12565019; doi:10.3390/jcm14207160)
Supplement: Supplementary file 1 [file jcm-14-07160-s001.zip › jcm-3830301-supplementary.pdf]

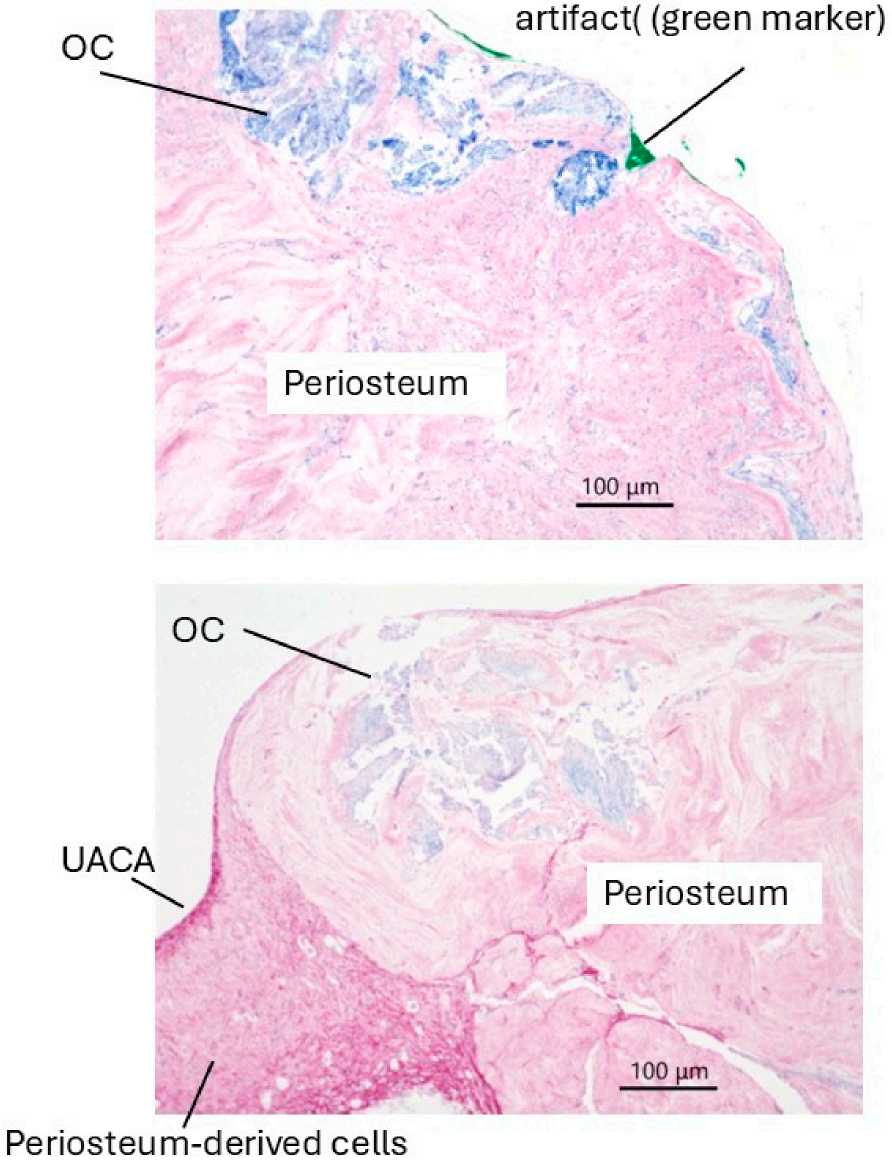

Figure S1. Double immunostaining of osteocalcin (OC: blue) and uveal autoantigen with coiled-coil domains and ankyrin repeats (UACA: red). The results of double immunostaining for OC and F-box and WD-40 domain-containing protein 2 (FBXW2) have been published in reference [26].

Table S1. Detected proteins and their regions in previous studies [17,18,26,27].

ESI-Q-TOF MS/MS and immunohistochemistry
Periosteal-derived cells and extracellular matrix: FBXL14, type I and III collagens
Capillaries of the periosteum: FBXL14
Elastic fibers in periosteum and blood vessels: FBXW2
Immunohistochemistry
Periosteum after explant culture: osteocalcin
ESI-Q-Orbitrap MS   jPOSTrepo (Japan ProteOme STandard Repository)
Suggested presence of type IV collagen
